# The ecological–evolutionary interplay: density-dependent sexual selection in a migratory songbird

**DOI:** 10.1002/ece3.254

**Published:** 2012-05

**Authors:** Thomas B Ryder, Robert C Fleischer, W Greg Shriver, Peter P Marra

**Affiliations:** 1 Smithsonian Conservation Biology Institute, Migratory Bird Center, National Zoological ParkPO Box 37012-MRC 5503, Washington, DC 20013-7012; 2 Smithsonian Conservation Biology Institute, Center for Conservation and Evolutionary Genetics, National Zoological ParkPO Box 37012-MRC 5503, Washington, DC 20013-7012; 3 Department of Entomology and Wildlife Ecology, University of DelawareNewark, Delaware 19716-2160

**Keywords:** Density, extra-pair paternity, sexual selection, urbanization

## Abstract

Little is understood about how environmental heterogeneity influences the spatial dynamics of sexual selection. Within human-dominated systems, habitat modification creates environmental heterogeneity that could influence the adaptive value of individual phenotypes. Here, we used the gray catbird to examine if the ecological conditions experienced in the suburban matrix (SM) and embedded suburban parks (SP) influence reproductive strategies and the strength of sexual selection. Our results show that these habitats varied in a key ecological factor, breeding density. Moreover, this ecological factor was closely tied to reproductive strategies such that local breeding density predicted the probability that a nest would contain extra-pair offspring. Partitioning reproductive variance showed that while within-pair success was more important in both habitats, extra-pair success increased the opportunity for sexual selection by 39% at higher breeding densities. Body size was a strong predictor of relative reproductive success and was under directional selection in both habitats. Importantly, our results show that the strength of sexual selection did not differ among habitats at the landscape scale but rather that fine-scale variation in an ecological factor, breeding density, influenced sexual selection on male phenotypes. Here, we document density-dependent sexual selection in a migratory bird and hypothesize that coarse-scale environmental heterogeneity, in this case generated by anthropogenic habitat modification, changed the fine-scale ecological conditions that drove the spatial dynamics of sexual selection.

## Introduction

The effect of ecological change on evolutionary processes and their dynamic interplay remains a fundamental conceptual focus in science (Schoener 2011). Moreover, while the relative role of ecological factors on evolutionary processes is fairly well established for some taxa (e.g., plants), our understanding of how environmental heterogeneity impacts phenotypic selection in most animal populations is limited (Garant et al. 2007; Kasumovic et al. 2008; Parker et al. 2011). Quantifying these relationships is particularly pertinent given recent evidence that human activities can alter evolutionary processes (Shochat et al. 2006; Smith and Bernatchez 2008). Of particular utility will be environment-specific estimates of selection that can quantitatively assess the spatial scale of adaptation and the mechanistic role of ecological variation on evolutionary processes (Svensson and Sinervo 2000, 2004). Despite the fundamental importance of an ecological–evolutionary interplay and evidence that the environmental heterogeneity associated with anthropogenic habitat modification will affect the direction and strength of selection (Smith et al. 2008), few studies have documented how selection varies for species that persist in human-dominated landscapes (Smith and Bernatchez 2008).

To date, urban ecology research has primarily focused on changes in community structure and the impacts of anthropogenic activities on ecological processes (McDonnell and Pickett 1990; Grimm et al. 2008). Moreover, while our understanding of the mechanisms that decouple fundamental patterns and evolutionary processes in human-dominated landscapes are in their infancy, they have begun receiving substantial attention (Shochat et al. 2006). For example, recent empirical work has documented that the novel selection pressures associated with urban environments can result in behavioral modifications (Slabbekoorn and Peet 2003), morphological divergence (Yeh 2004; Smith et al. 2008), divergent mate choice and reproductive tactics (Candolin et al. 2007; Perlut et al. 2008), and changes in population genetic structure (Bjorklund et al. 2010). Despite these advances no study, to our knowledge, has quantified how human habitat modifications and the ecological factors associated with these changes affect the dynamics of selection on phenotypic traits.

Here, we focus on sexual selection, which since its inception has largely been viewed as a subset of natural selection driven by competition over mates and subsequent variance in reproductive success (Andersson 1994). Sexual selection can operate via female choice (intersexual) and/or male–male competition (intrasexual) and is well established as the primary mechanism through which conspicuous dimorphism in secondary sexual traits arise (Andersson 1994). The opportunity for sexual selection to act is proportional to the variance in mating success (Wade and Arnold 1980; Arnold and Wade 1984a), and recent evidence suggests that both the strength of selection and the expression of secondary sexual traits can vary both spatially and temporally (Gosden and Svensson 2008; Cornwallis and Uller 2010). Traditionally, male reproductive success was measured as the number of social mates a male acquires during the breeding season (Webster et al. 1995; Whittingham and Dunn 2005), yet additional sources of variation can arise from biased operational sex ratios in which not all males obtain partners (Price 1984; Dearborn et al. 2001) and from differences in female quality such that some females produce more offspring than others (Kirkpatrick et al. 1990). A third source of variation stems from evidence that social and genetic mating systems are often incongruent, especially in birds, with some males siring offspring in broods raised by other males (Hasselquist and Sherman 2001; Griffith et al. 2002). The offspring resulting from these extra-pair fertilizations could potentially have considerable effect upon variance in mating success and subsequent sexual selection, since they increase the success of one male while simultaneously decreasing the success of another (Westneat et al. 1990). As such, the actual reproductive success of males in socially monogamous species is the combined total offspring sired from both within-pair and extra-pair fertilizations (Webster et al. 1995).

Given that ecological constraints (i.e., spatial and temporal distribution of mates) have long been thought to impose limits on the degree of sexual selection (Emlen and Oring 1977), here we ask how that relationship is modulated by anthropogenic habitat change. Specifically, we use a two-year study of ecology and paternity in the gray catbird (*Dumatella carolinensis*) to quantify reproductive strategies and the strength of sexual selection on phenotypes important in male–male competition and female mate choice in a human-dominated landscape. Gray catbirds were ideal for this study because they are a socially monogamous species that persists along the urbanization gradient yet show substantial variation in abundance among habitat types. Moreover, catbirds breed in a variety of anthropogenically modified habitats (i.e., the suburban matrix [SM] and suburban parks [SP]) that vary in habitat quality and breeding density. In particular, density has been shown to explain intraspecific variation in extra-pair paternity (Gibbs et al. 1990; Gowaty and Bridges 1991; Lifjeld et al. 1991; Yezerinac et al. 1999; Stewart et al. 2010) and the strength of selection (Bonenfant et al. 2003; Kokko and Rankin 2006; Gosden and Svensson 2008). Overall this study aims to understand how anthropogenic habitat modifications create a cascade of effects on ecological factors, reproductive strategies, and the adaptive value of traits under selection.

## Methods

### Study species and site

Our research focused on the gray catbird, a medium-sized migratory passerine (ca. 35 g) bird in the family Mimidae that breed from Canada to the southeastern United States and winters along the Gulf coast to the Caribbean and south to Panama (Cimprich and Moore 1995). Throughout their range catbirds are found breeding in dense shrubs or vine tangles and are often associated with early seral stage successional habitats (Zimmerman 1963; Peck and James 1987). These habitat preferences make catbirds common residents in both the matrix and suburban parks, yet breeding density varies markedly among our study sites with the matrix having lower density than parks (see Results). The catbird mating system is traditionally described as social monogamy (Cimprich and Moore 1995) although polygyny has been reported in two separate instances (Johnson and Best 1982). Like many other monogamous species, catbirds lack distinct plumage dimorphism although males tend to average more reddish edging on the undertail coverts (Suthers and Suthers 1990). Although catbirds lack apparent sexual plumage signals they are known to exhibit sexual size dimorphism (Raynor 1979; Lent 1990), with males being approximately 3% larger than females in several morphological measures.

We conducted this research from May to August in both 2008 and 2009 at five study sites (two SM sites and three SP sites embedded within the matrix) located in the greater metropolitan area of Washington, DC and Newark, Delware. Our SM sites were located in Takoma (35.3 ha) and Silver Spring, Maryland (18.7 ha), which had human population densities of 1308.9 and 1249.2/km^2^, respectively (United States Census Bureau 2000). Our SP sites were located at Sligo Creek Stream Park (9.0 ha), Wheaton Regional Park (19.9 ha), and the University of Delaware Ecology Woods (16.2 ha) located in Takoma Park and Wheaton, Maryland and Newark, Delaware, respectively. Matrix sites were comprised of individual properties within suburban neighborhoods that varied in size and landscaping but were broadly characterized by a broken canopy of large hardwood trees (oaks, *Quercus*; maples, *Acer*; and sycamores, *Platanus*) and yards that contained a mix of native and nonnative shrubs suitable for nesting. In contrast, SPs represented larger contiguous patches of habitat embedded within the SM characterized by a closed canopy of hardwoods and a continuous understory of dense and largely nonnative shrubs (e.g., multiflora rose and Japanese honeysuckle).

### Field methods

During both years of study, we captured adult catbirds using both systematic netting and target netting. Each captured individual was banded with a unique combination of colored leg bands and an aluminum band from US Fish and Wildlife Service (USFWS). At the time of capture a small blood sample (20–50 µL) was taken from the brachial vein and preserved in lysis buffer (Longmire et al. 1988) for paternity analyses. Detailed morphological measurements including wing, tail, tarsus (mm), and mass (g) were collected at four of the five sites (Delaware excluded) by a single measurer to minimize variation. Nests were located using parental behavior and systematic searches of the habitat. We visited each territory until we located an active nest and rotated among plots to ensure equal sampling effort. Nest contents were monitored every—two to four days and nestlings were banded with USFWS aluminum bands and bled (12–15 µL) two to three days prior to fledging. Despite differences in habitat types, nest survival (DSR, daily survival rate) did not differ significantly between study plots (χ^2^= 0.52, df = 3, *P*= 0.914) or among habitat types (χ^2^= 0.05, df = 1, *P*= 0.814; T. B. Ryder, unpubl. data). Social parents of each nest were primarily determined using target netting at each nest; however, identity of adults was confirmed using observations of parental feeding.

### Genetic analyses

DNA was extracted and purified using a Qiagen BioSprint 96 robotic system and DNeasy blood and tissue kit (Qiagen Inc., Valencia, CA, USA). We screened 16 microsatellite primers developed from Northern mockingbird, *Mimus polyglottos* (Hughes and Deloach 1997) and gray catbird (Cabe et al., unpubl. data) and optimized the six most polymorphic of these loci for genotyping. Polymerase chain reactions (PCRs) were run in 10 µL volumes consisting of 30 ng of genomic DNA, 1 µL of dNTPs (1mM deoxynucleotide tripohosphates), 1 µL of 5× Taq buffer (2.0 µM of MgCl_2_, 10 mM of Tris-HCl, 50 mM of KCl), 1 µL of 25 mM MgCl_2_, 0.5 µL of forward and reverse primers, 1 µL of 2.5× bovine serum albumin (BSA), and 0.1 µL of FlexiTaq polymerase (Promega Corp., Madison, WI, USA). PCR products were tagged using fluorescently labeled forward primers (Applied Biosystems, Inc., Carlsbad, CA, USA). PCR conditions consisted of initial denaturing at 94°C for 2 min, followed by 30–40 cycles of denaturation at 94°C for 30 sec, X°C annealing for 15–30 sec, extension at 72°C for 30 sec, followed by a final extension at 72°C for 10 min. The annealing temperature (X) was 50°C for DCA22 and DCD9, 55°C for DCC24, MP24, and MP25, and 57°C for DCD22. PCR products were pooled and loaded on a ABI 3100xl automated capillary sequencer together with an internal ladder (ROX 500 bp, Applied Biosystems, Inc.). Fragment sizes and genotypes were assigned using Genemapper 4.1 (Applied Biosystems, Inc.).

Each individual was typed at six microsatellite markers that varied in the number of independently assorting alleles and polymorphic information content (Table 1). We determined genotypes for 98% of the total possible cells, then calculated allelic frequencies and tested for linkage and departures from Hardy–Weinberg equilibrium using Genepop (Rousset 2008). None of the loci were linked or out of Hardy–Weinberg equilibrium and all loci showed low frequencies of both null alleles and typing error (Table 1). The six polymorphic loci yielded a high combined probability of exclusion for the paternity analyses (*P*_et_= 0.99).

**Table 1:** Details of the six polymorphic microsatellite markers used for gray catbird paternity analyses.

Locus	K^1^	*N*^2^	Hobs	Hexp	PIC^3^	Exc^4^	H–W^5^	FNull	Error^6^
DCA22	41	956	0.955	0.951	0.948	0.819	ns	–0.002	0.012
DCC24	30	953	0.915	0.914	0.907	0.706	ns	–0.001	0.011
DCD9	37	946	0.930	0.946	0.943	0.802	ns	+0.008	0.003
DCD22	13	956	0.871	0.876	0.863	0.599	ns	+0.002	0.033
MP24	20	955	0.865	0.885	0.874	0.623	ns	+0.012	0.026
MP25	28	890	0.939	0.942	0.938	0.787	ns	+0.001	0.007

1 Number of independently assorting alleles.

2 Number of individuals typed.

3 Polymorphic information content.

4 Exclusion probability of the first parent.

5 Hardy–Weinberg equilibrium tests.

6 Error rates calculated from known mother–offspring comparisons.

To minimize assigning offspring that match males by chance, we used the maximum-likelihood approach available in the program CERVUS v. 3.03 (Marshall et al. 1998; Kalinowski et al. 2007). Our preliminary simulations used 10,000 cycles and the true typing error (0.015), measured via known mother–offspring comparisons. We used females observed at the nest as putative mothers unless they had greater than one mismatch in which case the mother was assumed unknown. Offspring were assigned using both strict 95% and relaxed 80% confidence as well as a total evidence approach (Prodohl et al. 1998; Webster et al. 2004). Under all three scenarios, paternity was only assigned if the presumptive father matched the offspring at five or more loci. Using the total evidence approach, we assigned offspring only if other nestlings in the same brood were assigned with confidence to a sire known to be the attending male at the nest.

### Statistical analyses

We assessed local breeding density (hereafter just breeding density) as a potential ecological factor that could influence rates of extra-pair paternity and sexual selection. Specifically, we used nest locations as a proxy for breeding density (pairs/ha). We recorded nest locations (removing renests to avoid overestimating density) for all breeding pairs within the study sites using a Garmin etrex Vista HCx and mapped those locations in ArcMap V10 (ESRI, Redlands, California). To account for fine scale spatial differences in the density of breeding pairs, we generated a kernel density surface using the spatial analyst extension in ArcMap and the point layer of nests as our input. We then generated a continuous measure of density to be used in subsequent analyses by extracting an individual density estimate for each male from the kernel surface. We compared breeding density across sites using an analysis of variance (ANOVA) and used binary logistic regression to determine if density influenced rates of extra-pair paternity.

We calculated the opportunity for sexual selection in the two habitat types (SM and SP) as the standardized variance in male fertilization success following Webster et al. (1995). The standardized variance divided by the square of mean success is a measure of the maximum possible strength of selection (Arnold and Wade 1984a). We first calculated variance in apparent male success (var *T_A_*, the number of social young produced) and variance in realized reproductive success (var *T*, the number of offspring sired based on molecular paternity). We second partitioned total variance in realized male success into its component parts of within-pair (var(*W*)) and extra-pair fertilizations (var(*E*)) as well as the covariance (cov (*W*, *E*)) between the two to determine the effect of extra-pair young on the opportunity for sexual selection: var (*T*) = var(*W*) + var (*E*) + 2 cov(*W*, *E*) (Webster et al. 1995). Finally, given that comparisons of variance (apparent vs. relative) can be misleading about the effect of extra-pair paternity on the opportunity for sexual selection (Webster et al. 2007), we calculated a Batemans gradient (sexual selection gradient; Arnold 1994) by regression the number of mates against male reproductive success. The slope of this relationship provides a measure of the intensity of sexual selection arising from mating with additional females (extra-pair mates) outside the pair bond socially monogamous species (Arnold 1994; Arnold and Duval 1994).

We assessed sexual selection by regressing the number of offspring sired against male morphological traits (wing, tail, tarsus, mass, and body size) using stepwise least squares regression. We first used principle component analysis (PCA) to collapse the four morphological measurements into a single measure of overall body size. The largest portion of variance in male body size (45.2%, eigenvalue = 1.81) was accounted for by wing and tail, both of which loaded positively on the first principle component (PC1). Tarsus and weight loaded positively on PC2, which explained an additional 27.3% of the variance (eigenvalue = 1.09). Both axes were used as predictors in analyses. Second, we calculated linear selection differentials (β) for each habitat using regression coefficients (Lande and Arnold 1983). To ensure our selection differentials were comparable to previous work, we used standardized trait values (mean = 0, unit variance) and relative reproductive success (number of offspring sired/population mean). If individuals were present in more than one year we used mean breeding density and cumulative relative fitness over both years of study to avoid pseudoreplication in the selection analyses. Given the continuous nature of our fitness metric, significance of the selection coefficients were assessed using the least-square regression analyses. Fitness differentials were visualized by fitting relative fitness against traits of interest using a cubic spline model and smoothing parameter selected via generalized cross-validation (GCV) (Schluter 1988). Cubic spline models that minimized GCV were fit using a generalized additive model in program R using the mgcv package (R Team 2008; Wickham 2009). The contour surface was estimated using a Loess-smoothing algorithm with polynomial regression in Sigmaplot v.10.0.

To examine how selection varied by habitat type and an ecological covariate, we conducted two analyses using a generalized linear model framework. Generalized linear models used a normal error distribution, and identity link function and the maximum likelihood method for estimating model parameters. First, we added habitat as a categorical variable into the model and examined the interaction between significant morphological predictors of male reproductive fitness from previous analyses to determine the importance of anthropogenic habitat modification on selection dynamics. We specifically asked if the selection differentials observed in parks were different from those observed in the matrix by comparing the slopes of the two lines. Second, we examined both coarse-scale habitat and the fine-scale ecological covariate by using both habitat context (categorical) and breeding density (continuous; pairs/ha) as predictors in the model. Again, we were interested in the interaction between density and traits under selection. Finally, we compared the morphology of successful extra-pair sires against the social males of those nests in which the extra-pair young were present using paired *t*-tests. All statistics were done using JMP v.8.0.1 and means and standard errors are reported.

## Results

### Patterns of extra-pair paternity and breeding density

**Figure 1:**
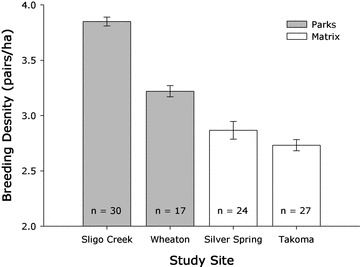
Breeding density (pairs/ha) by study site and habitat as measured using a kernel density surface. Suburban parks (SP) on average tended to have more breeding pairs than the suburban matrix (SM).

Over two breeding seasons, we sampled a total of 455 offspring from 165 broods at our five sites (Table 2). Of the total offspring we assigned paternity to 425 (93.4%), of which 401 (88%) were assigned at 95% strict confidence, 15 (3.3%) were assigned at 80% relaxed confidence, nine (2.0%) were assigned using total evidence, and the remaining 30 (6.6%) went unassigned. Genetic analyses revealed that 13% of catbird nestlings were the result of extra-pair fertilizations (all 95% strict confidence assignments) and that 24.8% of broods contained extra-pair young (EPY) (Table 2). We found no differences in the rates of extra-pair paternity (EPP) among years (2008 = 24.2%, 2009 = 26.4%; χ^2^= 0.53, df = 1, *P*= 0.46) or study populations (χ^2^= 0.81, df = 4, *P*= 0.94; see Table 2). In addition, we found no significant differences in EPP rate by habitat type (χ^2^= 0.76, df = 1, *P*= 0.38) despite parks having a slightly higher percentage of broods with EPY (26.17% vs. 22.45%). We next examined density as a potentially important ecological factor influencing rates of extra-pair paternity. The study sites showed significant variation in breeding density with parks on average having higher density than the matrix (*F*_3,94_= 3.85, *P*= 0.01; Fig. 1). Breeding density, as measured by the number of pairs/ha, had a significant positive effect on the occurrence of extra-pair paternity (χ^2^= 4.00, df = 1, *P*= 0.04, odds ratio = 0.84).

**Table 2:** Population-level patterns of extra-pair paternity estimated for gray catbirds nesting in the suburban matrix (SM) and suburban park (SP) sites in greater Washington, DC.

Site	Habitat type	No. of broods	No. of offspring	Percentage broods EPY (*N*)	Percentage EPY (*N*)
Silver Spring	SM	22	62	22.7 (5)	9.7 (6)
Takoma	SM	36	103	22.2 (8)	12.6 (13)
Sligo Creek	SP	33	86	30.3 (10)	12.8 (11)
Wheaton	SP	31	89	22.6 (7)	13.5 (12)
UD Ecology	SP	43	115	25.6 (11)	14.8 (17)
Total		165	455	24.8 (41)	13.0 (59)

### Opportunity for sexual selection

**Figure 2:**
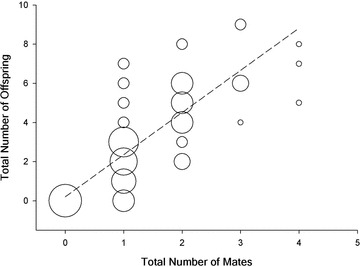
The Bateman's gradient for male gray catbirds, *Dumatella carolinensis*, showing the relationship between the number of genetic partners each male had and his reproductive success. Circles are sized proportional to sample size (small circles *n*= 1, largest circle *n*= 76).

Realized reproductive success of male gray catbirds ranged from—zero to nine offspring (mean = 2.03 ± 0.15) and did not significantly differ by habitat context (*t*= 0.62, df = 213, *P*= 0.53). Likewise, there were no differences between the matrix and parks in the number of within-pair (*t*= 0.97, df = 213, *P*= 0.33) and extra-pair (*t*=–1.18, df = 213, *P*= 0.24) offspring males sired. Males that sired extra-pair offspring, however, had higher reproductive success in both habitats (matrix, *t*= 3.00, df = 42, *P*= 0.004; parks, *t*= 5.03, df = 78, *P* < 0.0001). The relationship between extra-pair fertilizations and male fitness was further evidenced by a significant relationship between the total number of offspring and the total number of partners (*F*_1,214_= 460.73, *P* < 0.0001, *r*^2^= 0.68; Fig. 2). Partitioning the total opportunity for selection into its component parts revealed that variance in within-pair success (var *W* 86–91%) was the greatest contributor to realized male reproductive success in both environments (Table 3). Variance in extra-pair success (var *E* 7–14%) explained a smaller portion of standardized variance, although the extent varied by habitat (Table 3). Covariance explained a very small portion of the variance in male reproductive success in both habitats but was positive in the matrix and close to zero in park environments (Table 3). While we failed to find statistical differences in EPP rates among habitats, parks had substantially higher total standardized variance resulting in a 39% increase in the overall opportunity for sexual selection.

**Table 3:** The opportunity for sexual selection varies for gray catbirds breeding in two different ecological contexts. Standardized variances in apparent (number of social young produced, var (*T_A_*)) and realized reproductive success (number of offspring sired based on molecular paternity, var (*T*)) were calculated and weighted according to Webster et al. (1995). Components of variance were partitioned to determine the contribution of within-pair reproductive success (*W*), extra-pair reproductive success (*E*), and the covariance between the two (*W*, *E*) across two breeding seasons.

	Suburban matrix (*N*= 69)		Suburban park (*N*= 146)	
	Standardized value^1^	Total percentage	Standardized value^1^	Total percentage
var (*T_A_*)	0.872		1.27	
var (*T*)	0.826	100.0	1.15	100.0
var (*W*)	0.750	90.8	0.991	85.7
var (*E*)	0.059	7.1	0.163	14.2
cov (*W*, *E*)	0.017	2.1	0.001	0.10

1 Variance/mean^2^.

### Selection differentials

**Figure 3:**
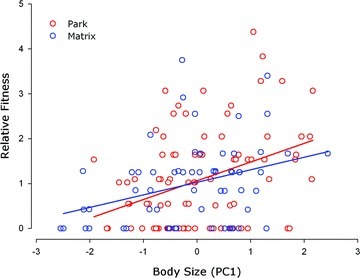
The relative male mating success of gray catbirds in relation to body size (PC1) in the suburban matrix (SM) (blue point and line) and suburban parks (SP) (red points and line) in greater Washington, DC. Selection differentials (β) show that body size is under strong directional selection in both habitats but the slopes of the lines are not significantly different.

**Figure 4:**
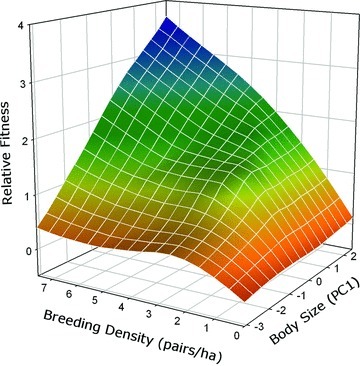
Relative fitness in relation to body size (PC1) and breeding density (pairs/ha) among male gray catbirds breeding in the suburban matrix (SM) and suburban parks (SP) in greater Washington, DC. The contour surface was estimated using a Loess-smoothing algorithm with polynomial regression.

In a step-wise regression, male body size (PC1) was the best predictor of relative fitness with larger bodied males siring more offspring than smaller individuals (*F*_1,151_= 23.43, *P* < 0.0001, *r*^2^= 0.14). We subsequently examined the selection differentials for male body size in each habitat. While male body size was under strong directional selection in both SP and the SM sites (β_park_= 0.42 ± 0.11, *F*_1,87_= 14.89, *P*= 0.0002, *r*^2^= 0.15; β_matrix_= 0.28 ± 0.09, *F*_1,60_= 8.73, *P*= 0.004, *r*^2^= 0.13; Fig 3), we found no evidence that the strength of selection differed between habitats (i.e., slope of lines) (χ^2^= 0.32, df = 1, *P*= 0.57; Fig. 3). Moreover, a second analysis showed that body size (χ^2^= 14.4, df = 1, *P*= 0.0001), breeding density (χ^2^= 92.3, df = 1, *P* < 0.0001), and their interaction (χ^2^= 7.6, df = 1, *P*= 0.0058) all influence a male's relative fitness while habitat context does not (χ^2^= 0.18, df = 1, *P*= 0.67). These results and an examination of a fitness surface indicate that large-bodied males in the highest breeding density environments had the highest relative fitness regardless of habitat context (Fig. 4). Finally, sexual selection for larger-bodied males was further evidenced by significant differences in body size (PC1) (Table 4) when making pair-wise comparisons between extra-pair sires and the within-pair males they cuckolded.

**Table 4:** Comparison of male gray catbird morphological traits of extra-pair sires and the males they cuckolded.

Trait	*N*	Extra-pair sires (mean ± SE)	Cuckolded males (mean ± SE)	*P*^1^
Wing (mm)	23	90.57 ± 0.51	90.48 ± 0.47	0.90
Tail (mm)	22	95.14 ± 0.76	94.45 ± 0.63	0.49
Tarsus (mm)	17	27.93 ± 0.23	27.31 ± 0.18	0.04
Mass (g)	22	35.03 ± 0.28	34.39 ± 0.32	0.14
Body size (PC1)^2^	17	0.63 ± 0.21	–0.04 ± 0.17	0.02

1 *P*-values from paired *t*-tests.

2 Wing and tail accounted for the largest portion of variance in male body size.

## Discussion

Sexual selection is an important force in the evolution of male sexual signals (Andersson 1994). Moreover, while sexual selection studies are common in natural environmental contexts, little is known about how anthropogenic activities affect reproductive strategies and subsequent selection pressures when species persist in human-dominated landscapes. Here, we document how the reproductive strategies of gray catbirds varied within two human-dominated habitats. In addition, we measured the relative opportunity for sexual selection and estimated selection differentials for traits related to male fitness. Our results show that rates of extra-pair paternity did not differ by habitat (matrix vs. park) but, that a finer spatial scale ecological variable, local breeding density influenced the probability of gaining extra-pair fertilizations and relative fitness. Partitioning reproductive variance showed that while within-pair success was the largest contributor to a male's reproductive success in both habitat contexts, extra-pair fertilizations explained a higher proportion of variance in the park habitats. Examining which male traits were under selection revealed that larger-bodied males have higher relative reproductive success but that the strength of selection was density dependent. Specifically, our fitness surface shows a peak for large-bodied males at the highest breeding densities. These results underscore that estimates of selection may be biased if research is only conducted in “high-quality/high-density” habitats and that studies should account for how selection dynamics are influenced by ecological factors.

### Patterns of extra-pair paternity and breeding density

Since the discovery that a substantial number of socially monogamous species exhibit sexual promiscuity (extra-pair paternity), biologists have put forward adaptive (Westneat and Sherman 1997; Petrie and Kempenaers 1998; Griffith et al. 2002) and nonadaptive hypotheses (Arnqvist and Kirkpatrick 2005; Forstmeier et al. 2011) to explain intra- and interspecific variation in EPP rates. To date, the majority of work at both taxonomic levels has focused on ecological factors that can impact the spatial and temporal availability of mates yet few studies have examined how these processes are modulated by anthropogenic habitat change (i.e., fragmentation and urbanization; but see Kasumovic et al. 2008; Townsend et al. 2011). Overall, our results show that gray catbird populations breeding in the matrix and parks have moderate levels of extra-pair paternity (25% of broods, 13% of offspring). While we failed to find statistical differences in EPP rates among populations or habitat type, breeding density did influence the probability of a brood containing extra-pair offspring in both habitat types. These results suggest that fine-scale ecological differences within habitat type can affect reproductive opportunities.

These results are also consistent with comparative evidence that density can play an important role in explaining EPP variation at the intraspecific level (Westneat and Sherman 1997; Moller and Ninni 1998; Griffith et al. 2002). Although we observed a relationship between density and EPP in our catbird populations, the relationship need not be causal if other factors that impact EPP rates and breeding density covary. For example, breeding density may be related to habitat structure such that male mate guarding is less efficient in the high shrub density environments of park habitats (Westneat and Sherman 1997). Higher breeding density may also reflect habitat quality and food availability such that males breeding in those environments are “emancipated” from a certain degree of parental care and can more readily seek extra-pair copulations (see Hoi-Leitner et al. 1999). Alternatively, extra-pair behavior may be constrained in urban landscapes because of the costs associated with seeking extra-pair mates (i.e., greater distances between territories; Norris and Stutchbury 2001). Regardless of the exact mechanism, individuals breeding in higher density environments experience higher mate encounter rates and are more likely to engage in extra-pair behaviors.

### Opportunity for sexual selection

In socially monogamous taxa, differential reproductive variance is largely driven by rates of extra-pair paternity, which can vary among populations based on ecological or behavioral factors (Griffith et al. 2002). Given that the strength of sexual selection is thought by some to be proportional to variance in mating success (Wade and Arnold 1980; Arnold and Wade 1984b; but see Klug et al. 2010a), extra-pair fertilizations, should theoretically increase the opportunity for selection (reviewed in Whittingham and Dunn 2004). Moreover, while there is little doubt that EPP can increase the opportunity for sexual selection, the magnitude of its effect appears to vary significantly among species (Freeman-Gallant et al. 2005; Whittingham and Dunn 2005; Albrecht et al. 2007; Dolan et al. 2007). In particular, estimating the effect of extra-pair success on the opportunity for sexual selection may be sensitive to population sampling (i.e., the proportion of sires sampled; Freeman-Gallant et al. 2005). Our cumulative assignment rates for all offspring (93%) and the extra-pair subset (67%) suggest that we adequately sampled our study populations and that our estimates of the opportunity for sexual selection are largely unbiased.

A second possible challenge associated with understanding the effects of extra-pair paternity on the opportunity for sexual selection has been the reliance on comparisons of standardized variances (apparent vs. realized). Recent work suggests that variance comparisons can be misleading and that extra-pair paternity can contribute to the opportunity for selection even in the absence of differences between apparent and realized success (see Webster et al. 2007; Klug et al. 2010a; Krakauer et al. 2011; While et al. 2011). Here, our results show a strong positive relationship between the number of partners a male has and his reproductive success (Bateman's gradient; Fig. 2) despite negligible differences in apparent and realized variance. Thus, males increased their fitness by engaging in extra-pair behavior and mating with females outside the social pair bond. Ultimately, if males in a socially monogamous system can gain via extra-pair fertilizations and increase their fitness, then the traits associated with extra-pair success will be favored by sexual selection (Webster et al. 2007). In this way, the additional variance associated with extra-pair mating can contribute to the strength of sexual selection on specific male phenotypic traits.

To date, most studies of socially monogamous passerines have shown that standardized variance in male reproductive success is largely explained by within-pair success (Webster et al. 1995, 2001; Freeman-Gallant 2005; Whittingham and Dunn 2005; but see Dolan et al. 2007). Our results largely corroborate this finding in which within-pair mating success explains the majority of the standardized variance (86–91%) in both habitats. In contrast, extra-pair success explained a smaller portion of the variance (7–14%) although this varied by habitat type. In the higher breeding density park environment, extra-pair success explained considerably more of the standardized variance in male reproductive success and was, in large part, responsible for the subsequent 39% increase in the opportunity for sexual selection. While covariance explained a marginal portion of the variance, the positive values in the matrix suggest that males were able to gain extra-pair fertilization without the trade-off of losing offspring in their social nest. Such trade-offs (extra-pair gains = within-pair losses) are often used to explain the limited impact of extra-pair mating on the opportunity for sexual selection (Webster et al. 1995). In contrast to the matrix, covariance in the higher density park environment was close to zero. These results suggest that males in environments with higher male–male competition cannot concurrently maximize both within- and extra-pair success because of the potential trade-offs between mate guarding and engaging in extra-pair behavior.

### Sexual selection, habitat, and local breeding density

Sexual selection can operate on a phenotypic trait via female choice (intersexual) and or male–male competition (intrasexual) and is supported when a trait covaries with reproductive success (Price 1984; Andersson 1994). While traits under sexual selection vary widely among taxa, a number of studies have shown body size to be a target of sexual selection (Andersson 1994; Gontard-Danek and Moller 1999). Here, we report that body size was a predictor of a male's relative reproductive success (WPY and EPY) for catbirds breeding in both matrix and park sites. While male body size is clearly related to reproductive output in catbirds, we are currently unable to differentiate the relative roles of intra- versus intersexual selection on trait elaboration. On one hand, large body size could provide advantages during male–male competition contests as has been shown for red-winged blackbirds (Searcy 1979; Eckert and Weatherhead 1987). Alternatively, female preference for male size could evolve via direct phenotypic benefits in which larger males provide material advantages (e.g., territory quality and/or parental care) or via the indirect genetic coupling of preference and trait (e.g., Fisherian and indicator mechanisms; Andersson 1994). Regardless of the mechanism, body size is heritable in birds (Garnett 1981; Wiggins 1989) such that selection for this male phenotype will yield larger offspring that may have a survival advantage in both environmental contexts. Ultimately, if large male body size affects viability attributes such as survival and reproductive success it may be favored by both natural and sexual selection in catbird populations.

While sexual selection may be the most parsimonious explanation for our findings, an alternative hypothesis is that the observed relationship between body size and fitness is indirectly caused by variation in rearing environment (see Bolund et al. 2011). As mentioned earlier, if breeding density varies with habitat quality than differences in rearing environments could indirectly influence body size even in the absence of strong selection. This idea is indeed plausible, yet in addition to the strong correlation between relative fitness and body size, our results also show that body size influenced extra-pair mating opportunities. Specifically, extra-pair males were on average larger than the within-pair males they cuckolded. This second line of evidence is also consistent with hypothesis that sexual selection is acting on male phenotype. Ultimately, only methods that can decouple the relative roles of genetic and environmental variation on body size (e.g., animal model approaches) will determine if the relationship between body size and fitness is causal.

Here, we aimed to understand how habitat context and its ecological covariates influence the strength of sexual selection in gray catbirds. At our study sites, habitat and breeding density were largely confounded, with parks having high density and the matrix having low density. While both habitat types occur within human-dominated landscapes, all of our study sites fell well within the range of expected variation in breeding density (e.g., 0.3 to >10 pairs/ha; Harcus 1973). Our results suggest that habitat type does not influence the strength of selection, yet the limited overlap in breeding density between park and matrix environments may have influenced our ability to detect the effect of coarse-scale environmental differences. As suggested by Wade and Kalisz (1990) manipulations of the ecological agents that drive selection are required to fully understand the selection process. As such, to fully understand the relative roles of habitat and an ecological covariate, such as density, on the strength of sexual selection, an experimental manipulation would be required.

While it has long been established that breeding density (i.e., the spatial and temporal distribution of mates) can be an important force shaping animal mating systems (Emlen and Oring 1977), only recently has work begun addressing how density influences the spatial and temporal dynamics of selection (Kokko and Rankin 2006; Gosden and Svensson 2008). Thus far, results are mixed ranging from density having a strong impact on the strength of sexual selection (Conner 1989; Clutton-Brock et al. 1997; Bonenfant et al. 2003; Kasumovic et al. 2008; Lehtonen and Lindstrom 2008) to little or no effect (Head et al. 2008; Klug et al. 2010b). Our results show that male body size in gray catbirds was under the strongest selection in the highest breeding density environments. In contrast, lower density environments likely experienced relaxed sexual selection because of limited mate choice opportunities and mate acquisition (Candolin et al. 2007; Candolin and Heuschele 2008). Other recent empirical work has hypothesized that localized density- and frequency-dependent interactions between the sexes may emerge as a primary driver of spatial variation in sexual selection (Gosden and Svensson 2008). Viewed cumulatively our results support this hypothesis and suggest higher density environments increase the probability of extra-pair fertilizations, the opportunity for and the strength of sexual selection. More broadly, our results suggest that coarse-scale environmental heterogeneity, in this case generated by anthropogenic habitat modification, can influence the fine-scale ecological conditions that drive reproductive strategies and the spatial dynamics of sexual selection.
